# Home and Community-Based Services Between the USA and Taiwan

**DOI:** 10.1093/geroni/igab046.1349

**Published:** 2021-12-17

**Authors:** Xian Cao, Su-I Hou

**Affiliations:** 1 University of Central Florida, University of Central Florida, Florida, United States; 2 School of Global Health Management & Informatics, University of Central Florida, University of Central Florida, Florida, United States

## Abstract

Home and community-based services (HCBS) are critical to support our rapidly growing and aging population around the world. This paper takes initial steps to compare HCBS between the USA and Taiwan from four perspectives: funding sources, service types, challenges, and recommendations. Peer-reviewed articles and governmental reports (both English and Chinese) in the U.S.A. and Taiwan were reviewed. Analyses showed both countries mainly use tax dollars to fund HCBS. Although both countries have similar service categories, USA lack a clear organization scheme whereas Taiwan has detailed and clear services provisions. Workforce quality and shortage were common challenges for both countries, especially from culture perceptive. Recommendations for USA include expanded funding pool, better coordination between agencies, and rebalancing HCBS and institutional care with limited budget. Recommendations for Taiwan include expanded service coverage and quality to reduce disparity in rural areas, and providing more support for informal caregivers.

